# Prevalence of Eating Disorders and Self-Perception Concerning Body Composition Analysis among Elite Soccer Players

**DOI:** 10.5114/jhk/194464

**Published:** 2024-12-06

**Authors:** Wiktoria Staśkiewicz-Bartecka, Grzegorz Zydek, Małgorzata Magdalena Michalczyk, Marek Kardas

**Affiliations:** 1 Department of Food Technology and Quality Evaluation, Medical University of Silesia in Katowice, Katowice, Poland.; 2 Institute of Sport Sciences, Jerzy Kukuczka Academy of Physical Education in Katowice, Katowice, Poland.; 3 Department of Sport Nutrition, Jerzy Kukuczka Academy of Physical Education in Katowice, Katowice, Poland.

**Keywords:** eating habits, team sport, health, diet, athletes

## Abstract

In the field of mental health, eating disorders (EDs) are an important subject of research, especially regarding athletes. This study examined the risk of EDs, orthorexia nervosa (ON), and body perception among elite soccer players, focusing on the impact of body composition. Conducted from March to April 2024 with 51 players from the elite and 1^st^ division clubs, the research utilized the Eating Attitudes Test (EAT-26), the Düsseldorf Orthorexia Scale (DOS), and the Body Esteem Scale (BES) alongside body composition analysis via Direct Segmental Multi-Frequency Bioelectrical Impedance Analysis (DSM-BIA). Findings indicated a significant prevalence of ED risk among players, with more than half showing potential symptoms. Body composition analyses revealed that players with a higher fat mass had an increased ED risk, while muscle mass did not correlate significantly with ED attitudes. Regarding ON, approximately one-third of athletes were at risk, although no significant relationship was found between ON and body composition variables. In terms of body image, players generally viewed their bodies moderately, rating physical fitness, sex drive, and health positively. However, a higher body fat was associated with lower ratings in upper body strength, indicating perceived physical limitations. This study highlights the necessity for targeted interventions to manage the high prevalence of EDs among elite soccer players and promote healthier body image perceptions, emphasizing the low relationship between body esteem and the likelihood of developing EDs or ON.

## Introduction

In the field of mental health, eating disorders (EDs) are an important subject of research, especially regarding athletes. Nowadays, physical culture has a significant impact on eating habits and self-perception (Neglia, 2021). Eating disorders can stem from feelings of dissatisfaction with one's physical appearance, as well as a desire to lose weight to achieve better performance in sports (Marí-Sanchis et al., 2022; Pustivšek et al., 2020). Athletes are a particularly susceptible group. According to current studies, athletes experience EDs more often than the general population (Marí-Sanchis et al., 2022).

Cultural differences significantly influence the manifestation of EDs, as evidenced by extensive research. Societies that emphasize the thin-ideal, predominantly seen in Western cultures, exhibit higher rates of body dissatisfaction and ED behaviors (Smitch et al., 2020). Additionally, the relationship between acculturation and ED pathology has been extensively explored, revealing that cultural factors substantially impact the development of EDs. Factors such as acculturation, cultural change, and intergenerational conflict are notably associated with ED symptoms (Okumuşoğlu, 2018). Moreover, comparative research on individuals diagnosed with anorexia nervosa (AN) from Western and non-Western countries highlights distinct differences in psychopathological expression. Western societies display higher levels of body dissatisfaction and overall psychopathology compared to non-Western countries such as China (Gaspar et al., 2022). The cultural pressure to conform to thin ideals, coupled with the portrayal of ideal bodies in media, contributes to the complex relationship between cultural norms and the development of EDs (Melisse et al., 2020). These findings underscore the critical importance of considering cultural influences in the understanding and treatment of EDs.

Two major categorization systems for mental disorders have been used to classify EDs based on their respective criteria. The International Classification of Diseases and Health Problems (ICD-11) is used in European countries, while the Diagnostic and Statistical Manual of Mental Disorders (DSM-5) is used in the United States. Following these classifications, doctors can accurately diagnose and treat people with EDs including bulimia nervosa (BN), anorexia nervosa, and other related conditions (American Psychiatric Association, 2013; World Health Organization, 2019). However, the aforementioned classifications lack a disorder increasingly described in empirical studies, which is orthorexia nervosa (ON) (Koven and Abry, 2015).

ON is a condition characterized by an obsession with eating only foods perceived as healthy. A person with this disorder focuses on an extreme fixation on healthy eating, which leads to self-imposed dietary restrictions that can potentially result in somatic and psychosocial consequences (Costa et al., 2017; Grajek et al., 2022). ON can lead to anxiety, stress, and self-loathing due to the strict focus on food quality and the elimination of certain food groups from the diet. Studies indicate that ON can affect people of all ages, with a prevalence in the population ranging from 1% to 60% (Hayes et al., 2017).

Soccer is one of the team sports that is becoming increasingly popular at both amateur and professional levels, attracting players of all ages to the field (Kamienski, 2022). It is crucial to understand the possible risks to the physical and mental health of soccer players (Sander et al., 2021). Soccer players may experience body dissatisfaction due to many factors, such as comparisons with other athletes, social pressures or physical performance expectations (Ponce-Gonzalez et al., 2021; Woods et al., 2022). Idealized standards of physicality and beauty are often promoted on social media, which can encourage the obsessive pursuit of physical perfection (Baldó Vela et al., 2022; Mascherini et al., 2020). In addition, it should be noted that professional sports place a significant burden on both physical and mental health. The pursuit of excellence in professional sports can be dangerous, leading to various health consequences and an increased risk of mental disorders (Costa et al., 2017; Woods et al., 2022).

Recent literature suggests that the body mass index (BMI) is not a reliable indicator of body mass in athletes such as soccer players, since it does not distinguish between muscle mass and fat. About 17% of soccer players might be at risk of EDs, with the BMI not being a suitable measure due to their muscle mass (Carling and Orhant, 2010; Staśkiewicz-Bartecka and Kardas, 2024; Staśkiewicz et al., 2022). However, there are no studies in the current literature evaluating the risk of EDs and ON in a group of elite soccer players with consideration of body composition.

The purpose of this study was to assess the risk of EDs including ON and to evaluate self-perception of the body in a group of elite soccer players. In addition, the goal was to determine the impact of body composition on the prevalence of these disorders. This study aimed to identify potential mental health risks that may exist in this specific group of athletes.

The following working hypotheses were used in the study design: (1) the risk of eating disorders among elite soccer players is common, (2) players with lower body fat content are at a higher risk for these disorders, (3) players with higher body fat content and lower muscle mass show lower levels of satisfaction with their bodies.

## Methods

### Design and Procedures

The survey was conducted from March to April, 2024. The study included players of two soccer clubs, that participated in the elite and 1^st^ division and were located in the Silesian Agglomeration. The study used dedicated sampling. With this method, the sample was selected to represent characteristics and specific experiences related to the topic of the study. Determining precise selection criteria, such as gender, sports discipline, and the performance level was crucial to achieve the objectives of the study.

The survey was conducted in the first half of the competitive period of the spring round of the 2023/2024 season, all players were surveyed in the same period (between the 7^th^ and 8^th^ round of games) to obtain comparable results. A survey questionnaire was used to conduct the study, which consisted of a metric section and questionnaires of the Eating Attitudes Test (EAT-26), the Düsseldorf Orthorexia Scale (DOS, PL-DOS) and the Body Esteem Scale (BES), as well as body mass composition analysis by Direct Segmental Multi-Frequency Bioelectrical Impedance Analysis (DSM-BIA). Completion of the questionnaires by athletes and body composition measurements took place in the same week. Athletes completed the questionnaires at the club after training, and instruction was provided to participants before completing the questionnaires to ensure proper understanding and interpretation of the questions and to minimize completion errors. The response rate was 94.4%.

Participants were informed about the purpose of the study and its anonymity and were asked to accept the rules of data sharing. Information about informed and voluntary participation in the study was also provided at the beginning of the questionnaire. The World Medical Association's Declaration of Helsinki guided the conduct of this study. The study was approved by the Bioethics Committee of the Silesian Medical University in Katowice (approval code: BNW/NWN/0043-3/641/35/23; date of approval: 22 September 2023).

### Participants

The study included 51 soccer players, from two sports clubs participating at the elite (N = 26) and 1^st^ division (N = 25). Players were between the ages of 19 and 36, while the average age of respondents was 26.0 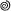 5.0. Players were of different nationalities (43 Polish, 2 Spanish, 2 Slovak, 1 Japanese, 1 Georgian, 1 British, and 1 Macedonian). The inclusion criteria were the following: (1) consent to participate in the study, (2) age 18 or older, (3) status of an active club player at the time of the study, (4) no injury that excluded the player from training for at least seven days in the last two months preceding the study, (5) knowledge of Polish or English, (6) no EDs identified in the past or currently, (7) no contraindications to body composition analysis. Exclusion criteria were as follows: (1) an incorrectly or incompletely completed questionnaire, (2) absence, (3) presence of contraindications to body composition analysis.

### BMI

Body height (cm) and body mass (kg) were measured with 0.1-cm and 0.1-kg precision (SECA 756, Seca gmbh & Co. KG., Hamburg, Germany) using the InBody 570 (InBody USA, Cerritos, CA, USA). The body mass index (BMI) was calculated by dividing the body mass (kg) by the square of the body height (m). The results were used as the basis for comparing height-to-weight ratios for the European population to WHO recommendations and guidelines (World Health Organization, 2010).

### DSM-BIA

Body composition was assessed using the DSM-BIA (Direct Segmental Multi-Frequency Bioelectrical Impedance Analysis) (InBody 570, InBody USA, Cerritos, CA, USA). The DSM-BIA method is based on the concept of dividing the human body into five cylindrical segments, allowing accurate measurement of the biological resistance, or impedance, of individual body parts. The test uses a system of tetrapolar and eight-point electrodes that separately measure the impedance of the torso, arms, and legs, using three different frequencies (5, 50, 500 kHz) for each segment and a current of 400 uA (InBody, 2024). Body composition analysis provided information on lean body mass (LBM), total body water (TBW), skeletal muscle mass (SMM), fat mass (FM), and fat mass percentage (%FM). The device manufacturer's normal protocol was followed when performing the measurements. The analyzer was tested using a calibration circuit of known impedance (resistance = 500.0 Ω; reactance = 0.1 Ω; error 0.9%) before each testing session (Kotler et al., 1996).

### EAT-26

The Eating Attitudes Test (EAT-26) created by Garner (2024) was utilized in the study as a screening tool to determine ED risk. It was created to test people who were at risk for obesity, AN, or BN as well as those already clinically diagnosed. Due to the presence of athletes of different nationalities in the study group, both the original version of the questionnaire and a Polish version of the tool (Włodarczyk-Bisaga and Dolan, 1996) were used. Three “referral criteria" were used to evaluate the results of the EAT-26 questionnaire and determine whether to submit a participant for additional ED risk assessment.

First, the final score on the EAT-26 questionnaire was considered. The EAT-26 consists of 26 questions about attitudes toward nutrition and scores are awarded as follows: for questions 1 through 25, one may receive 3 points for “always” response, 2 points for “usually”, 1 point for “often”, and 0 for other responses. In contrast, question 26 is graded differently as “never” response is awarded 3 points, etc. The exam's total score might vary from 0 to 78. A person with a score below 20 is thought to be at risk of developing an ED and ought to see a professional for a more precise diagnosis.

Second, inquiries concerning behavioral patterns may point to the existence of ED symptoms or a recent, notable weight loss. These questions focus on compensatory behavior which includes using laxatives, inducing vomiting, overeating, engaging in excessive physical activity, and losing weight at an increased rate. If the response to any of these questions is in the affirmative, it could indicate the existence of anomalies and the necessity of a further ED diagnosis.

Finally, if body mass is low when compared to age norms, the BMI may indicate ED risk. Potential dangers and the need for additional assessments are identified by evaluating the BMI about respondents' height, weight, and gender data.

### E-DOS, PL-DOS

The Düsseldorf Orthorexia Scale (DOS) is a tool for the initial assessment of eating behaviors associated with ON. The DOS is characterized by good internal consistency construct validity, and consistent reliability across time measurements, according to reference (Meule et al., 2020). The scale contains ten questions as part of a broader tool consisting of 21 items that branch into three subscales: eating behavior, avoidance of additives, and attention to mineral intake. The ten-item DOS questionnaire serves as a one-dimensional tool for screening and assessing ON (Barthels et al., 2015). Responses are given on a four-point Likert scale, starting from “strongly disagree” to “strongly agree”, with no questions in the negative form. The maximum possible number of points to be scored on the test is 40. The interpretation of the results is as follows: a total score above 30 suggests the presence of ON, a score between 25 and 29 indicates a potential risk of developing it, while a score below 25 indicates the absence of the disorder (Barthels et al., 2015). In the present study, due to the diversity of participants' nationalities, both the English version of the ten-item questionnaire assessing orthorexia (E-DOS) and its Polish translation (PL-DOS) were used. The study showed that the Polish version of the PL-DOS was a reliable counterpart to the English version, as evidenced by reliability indices (Cronbach's α = 0.840 and ω = 0.840) (Barthels et al., 2015; Brytek-Matera 2021).

### BES

To examine participants' body self-esteem, we used the Body Esteem Scale (BES), the Polish adaptation which was developed by Lipowska and Lipowski (Franzoi and Shields, 1984; Lipowska and Lipowski, 2013). The scale assesses respondents' attitudes toward their own bodies. Due to the multinationality of the participants in the current study, both the original version of this tool and its Polish version were used. The Polish adaptation has shown high reliability compared to the English-language version, achieving an α = 0.93 for all respondents and 0.94 for the male subgroup (Lipowska and Lipowski, 2013). The scale is comprised of 35 questions, which are divided into three subscales differentiated by respondents' gender. For men, the categories include physical attractiveness (PA), upper body strength (UBS), and physical condition (PC). Participants answer the questions using a five-point Likert scale, where “1” represents very negative feelings, “5” represents very positive feelings, and “3” represents a neutral point. In the analysis of the results, a normative table was used that takes into account different age categories and stenes, which allows adequate interpretation of the results obtained by men. Results falling between sten 1 and 3 indicate low body self-esteem, results in the range of 4–7 indicate medium self-esteem, while results reaching the range of 8–10 suggest high body self-esteem (Franzoi and Shields, 1984; Lipowska and Lipowski, 2013)

### Statistical Analysis

Statistical analyses were performed using Statistica v.13.3 (Stat Soft Poland) and the R package v. 4.0.0 (2020) under the GNU GPL (The R Foundation for Statistical Computing). To present quantitative data, mean values and standard deviations (X ± SD) were calculated; for qualitative data, the percentage notation was used.

Compliance with the normal distribution was checked using the Shapiro-Wilk test. The significance of differences between soccer players of different playing positions was assessed using analysis of variance (ANOVA) for three or more parametric groups and the Kruskal-Wallis test for three or more non-parametric groups. The chi-square test was used to analyze categorical data, such as the percentage of respondents at risk for EDs and ON based on EAT-26 and DOS questionnaire results. ANOVA was used to compare the EAT-26, DOS, and BES test results in different playing positions. The Fisher's exact test and the Chi-square test were used to analyze the relationship between EDs and ON risk and BES scores and a playing position. One-way ANOVA or the Kruskal-Wallis test was used to evaluate the associations between EDs, ON risk, BES scores, the BMI and mean body composition measurements. To examine the association between EAT-26, DOS, and BES scores, the chi-square test and the Cramer's V coefficient were used. The value of this coefficient made it possible to measure the strength of the relationship between two categorical variables, i.e., ED and ON risk scores and body composition scores.

A value of *p* < 0.05 was used as a criterion for statistical significance.

## Results

### Sample Characteristics

Fifty-one soccer players participated in the study after taking into account inclusion and exclusion criteria. The participants in the study had junior high school education (n = 2; 3.90%), high school education (n = 38, 74.50%) or higher education (n = 11, 21.60%). Two soccer players suffered from chronic diseases (Hashimoto's and asthma) and were taking constant medication (Euthyrox and Berodual). Statistically significant differences were found between players for various playing positions on the field. These differences were in body height (*p* < 0.001), body mass (*p* = 0.004), the BMI (*p* = 0.032), and body composition variables such as LBM (*p* < 0.001), TBW (*p* < 0.001), and SMM (*p* = 0.001). The characteristics of the study group are shown in Table 1.

**Table 1:** Characteristics of the study participants (N = 51); values presented as mean ± SD.

Variable	Total (n = 51)	Forwards(n = 8)	Midfielders (n = 23)	Defenders (n = 15)	Goalkeeperss (n = 5)	*p*-value
**Age [years]**	26.00 ± 5.00	28.00 ± 5.29	25.60 ± 5.02	26.00 ± 4.97	25.20 ± 5.31	0.752
**Height [cm]**	180.80 ± 6.78	181.6 ± 6.58	177.00 ± 5.55	183.90 ± 5.85	187.70 ± 3.07	<0.001*
**Body mass [kg]**	77.20 ± 8.20	79.70 ± 7.12	73.00 ± 8.64	81.30 ± 6.37	79.90 ± 3.82	0.004*
**BMI [kg/m^2^]**	23.50 ± 1.32	24.10 ± 0.82	23.20 ± 1.61	24.00 ± 0.87	22.70 ± 0.86	0.032*
**LBM [kg]**	69.40 ± 8.20	72.95 ± 7.32	64.93 ± 8.14	73.35 ± 6.64	72.42 ± 4.18	<0.001*
**TBW [kg]**	50.64 ± 5.91	53.17 ± 5.26	47.38 ± 5.90	53.50 ± 4.68	53.00 ± 3.00	<0.001*
**SMM [kg]**	39.92 ± 4.95	42.15 ± 4.53	37.22 ± 4.86	42.31 ± 4.02	41.64 ± 2.67	0.001*
**FM [kg]**	7.76 ± 2.23	6.75 ± 2.50	8.10 ± 2.48	7.88 ± 1.88	7.46 ± 1.46	0.156
**%FM [%]**	10.15 ± 2.87	8.51 ± 3.01	11.11 ± 3.11	9.81 ± 2.29	9.36 ± 1.81	0.623

* p < 0.05; BMI: Body Mass Index; LBM: Lean Body Mass; TBW: Total Body Water; SMM: Skeletal Muscle Mass; FM: Fat Mass; %FM: Fat Mass Percentage

The main source of nutritional knowledge for all players was a dietician (n = 41; 80.40%), followed by the Internet (n = 35; 68.60%). Soccer players were least likely to indicate a coach (n = 9; 17.60%) and friends (n = 9; 17.60%). The majority of respondents did not exclude any food group from their diet (n = 34, 66.7%). Soccer players most often excluded lactose-containing products (n = 4; 7.80%), dairy products (including those without lactose) (n = 3; 5.90%), gluten-containing grain products (n = 3; 5.90%), soy and related products (n = 3; 5.90%), one person did not eat meat and one athlete did not eat eggs. Athletes were also asked about performing additional training outside the sports club, and it was found that 94.10% of players (n = 48) also trained on their own. Most athletes performed 1–2 additional training units per week (n = 19; 38.80%), followed by 2–4 units per week (n = 11; 22.40%). At least one additional workout per week was indicated by 16.30% of respondents (n = 8), 14.30% of athletes performed additional training occasionally (n = 7), while 8.2% of athletes (n = 4) declared to perform additional training sessions 5–6 times per week. There were no statistical differences between playing positions and the amount of additional training (*p* = 0.323).

### Risk of EDs

Based on the result of the EAT-26, Part A questionnaire, it was estimated that 5.9% (n = 3) of respondents were at risk for eating disorders and should consult a specialist for further diagnosis. No significant differences were found between the playing position and the total EAT-26, Part A test score (*p* = 0.054). Significant differences were found between LBM (*p* = 0.028), TBW (*p* = 0.028), SMM (*p* = 0.031), and EAT test scores (Part A), and players with higher values had a lower risk of EDs. According to the results from the behavioral questions of the EAT-26 test, Part B, it was estimated that 47.1% (n = 24) of soccer players met the criterion indicating a risk of developing EDs. There was no significant effect of the position played on the field on the results of the EAT-26 test for behavioral questions (*p* = 0.880). However, there was a significant relationship between FM and the EAT score (part B), and players with higher FM had a higher risk of EDs (*p* = 0.028). No athlete's BMI with age norms indicated ED risk, as assessed by Part C.

Based on the overall results and interpretation of the EAT-26 questionnaire, 51% (n = 26) of the respondents met at least one of three criteria that indicate the likely existence or susceptibility to EDs and should consult a specialist for further diagnosis. There was no significant effect of the playing position on the overall EAT-26 score (*p* = 0.962). However, there was a correlation between FM and the overall test score, and players with higher FM presented a greater risk of EDs (*p* = 0.035). Detailed results are shown in Table 2.

**Table 2:** Summary of ED risk estimation (EAT-26) according to body composition (n = 51); values presented as mean ± SD.

Variable		EAT-A	*p*-value	EAT-B	*p*-value	EAT-26	*p*-value
**BMI [kg/m^2^]**	Risk	24.70 ± 0.64	0.078	23.86 ± 1.45	0.136	23.86 ± 1.42	0.118
	No Risk	23.48 ± 1.32		23.28 ± 1.16		23.26 ± 1.17	
**LBM [kg]**	Risk	79.80 ± 6.21	0.028*	70.42 ± 9.08	0.624	70.52 ±8.90	0.486
	No Risk	67.75 ± 7.91		68.49 ± 7.39		68.32 ± 7.48	
**TBW [kg]**	Risk	58.03 ± 4.43	0.028*	51.37 ± 6.56	0.637	51.44 ± 6.43	0.498
	No Risk	50.18 ± 5.71		49.99 ± 5.31		49.87 ±5.38	
**SMM [kg]**	Risk	46.43 ± 3.93	0.031*	40.51 ± 5.51	0.617	40.57 ± 5.40	0.492
	No Risk	39.52 ± 4.76		39.40 ± 4.44		39.30 ± 4.50	
**FM [kg]**	Risk	6.37 ± 2.07	0.289	8.40 ± 2.18	0.028*	8.36 ± 2.14	0.035*
	No Risk	7.85 ± 2.23		7.20 ± 2.16		7.19 ± 2.21	
**%FM [%]**	Risk	7.33 ± 2.36	0.063	10.79 ± 2.97	0.079	10.72 ± 2.93	0.111
	No Risk	10.32 ± 2.83		9.58 ± 2.70		9.60 ± 2.75	

* p < 0.05; BMI: Body Mass Index; LBM: Lean Body Mass; TBW: Total Body Water; SMM: Skeletal Muscle Mass; FM: Fat Mass; %FM: Fat Mass Percentage

According to the interpretation of the DOS questionnaire results, 33.3% (n = 17) of the players had the risk of ON, while the result of 7.8% (n = 4) of the subjects indicated the presence of ON. There was no statistically significant relationship between the risk or presence of ON and position on the field (*p* = 0.364) There was also no relationship between body composition variables and DOS score (Table 3).

**Table 3:** Summary of ON risk assessment (DOS) according to body composition (n = 51; values presented as mean ± SD).

DOS	BMI [kg/m^2^]	LBM [kg]	TBW [kg]	SMM [kg]	FM [kg]	%FM [%]
**No risk <25**	23.3 ± 1.28	68.1 ± 8.15	49.7 ± 5.89	39.1 ± 4.90	7.88 ± 1.99	10.5 ± 2.66
**Risk 25–29**	23.9 ± 1.41	70.8 ± 8.20	51.7 ± 5.90	40.9 ± 4.97	7.53 ± 2.33	9.72 ± 2.89
**Presence >30**	24.0 ± 1.15	73.2 ± 8.41	53.4 ± 6.02	41.1 ± 5.21	7.85 ± 3.90	9.52 ± 4.62
***p*-value**	0.210	0.119	0.181	0.132	0.543	0.299

BMI: Body Mass Index; LBM: Lean Body Mass; TBW: Total Body Water; SMM: Skeletal Muscle Mass; FM: Fat Mass; %FM: Fat Mass Percentage

### Attitude Towards One's Own Body

According to the BES results’ interpretation, soccer players were mostly characterized by moderate body evaluation in the categories of physical attractiveness (56.9%; n = 29), upper body strength (60.8%; n = 31), and physical fitness (58.8%, n = 30). There was no correlation between self-assessment of the body in all subscales and the playing position (PA, *p* = 0.395, UBS, *p* = 0.540, PC, *p* = 0.411). Analysis of body composition showed a significant relationship between UBS and %FM subscale self-assessment. Athletes with lower %FM had higher body assessments in this category (*p* = 0.047) (Table 4).

**Table 4:** Assessment of the athletes' body attractiveness according to BES interpretation concerning body composition variables (n = 90; values presented as mean ± SD).

Assessment of the attractiveness subscale		BMI [kg/m^2^]	LBM [kg]	TBW [kg]	SMM [kg]	FM [kg]	%FM [%]
**PA**	**Low**	23.8 ± 2.94	71.53 ± 15.9	52.17 ± 11.48	41.17 ± 9.4	8.18 ± 1.08	10.53 ± 2.16
	**Medium**	23.7 ± 1.22	69.44 ±8.59	50.66 ± 6.18	39.94 ± 5.23	8.01 ± 2.47	10.46 ± 3.14
	**High**	23.2 ± 0.99	68.86 ± 5.48	50.27 ± 4.0	39.61 ± 3.33	7.28 ± 2.0	9.56 ± 2.59
	***p*-value**	0.329	0.849	0.879	0.874	0.585	0.603
**UBS**	**Low**	23.72 ± 1.26	61.30 ± 9.46	44.70 ± 6.93	34.75 ±5.16	10.30 ± 3.68	14.10 ± 2.55
	**Medium**	23.57 ± 1.5	68.60 ± 8.3	50.06 ± 5.99	39.44 ± 5.02	8.17 ± 2.09	10.72 ± 2.62
	**High**	23.48 ± 0.92	72.29 ± 7.13	52.74 ± 5.12	41.72 ± 4.29	6.51 ± 1.87	8.33 ± 2.48
	***p*-value**	0.337	0.117	0.111	0.101	0.087	0.047*
**PC**	**Low**	24.83 ± 0.47	77.95 ± 4.60	56.75 ± 3.18	45.3 ± 3.54	6.35 ± 3.32	7.60 ± 4.1
	**Medium**	23.54 ± 1.51	67.29 ± 8.31	49.36 ± 6.01	38.84 ± 4.96	9.28 ± 2.1	10.96 ± 2.51
	**High**	23.43 ± 0.98	71.29 ± 7.59	52.02 ± 5.42	41.06 ± 4.6	7.09 ± 2.22	9.14 ± 2.97
	***p*-value**	0.287	0.058	0.054	0.059	0.255	0.115

* p < 0.05; BMI: Body Mass Index; LBM: Lean Body Mass; TBW: Total Body Water; SMM: Skeletal Muscle Mass; FM: Fat Mass; %FM: Percentage Fat Mass Percentage; PA: Physical Attractiveness; UBS: Upper Body Strength; PC: Physical Condition

Considering particular body features, the highest average scores were obtained for physical fitness (4.45 points), sex drive (4.43 points), health (4.52 points), and sexual activity (4.52 points). The least attractive to respondents were feet (3.65 points) and body hair (3.71 points).

### Correlation between the EAT-26 Score, DOS Score and BES

The relationship between the interpretation of the EAT-26 and DOS test scores and the interpretation of BES scores on all three subscales was analyzed. There was no significant association between the risk of developing EDs and ON and scores on the PA, UBS, and PC subscales (Table 5).

**Table 5:** Relationship between EAT-26, DOS, and BES scores.

	BES				
**EAT-26**	**Low**	**Average**	**High**	***p*-value**	**V Cramer**
	**PA**				
**No risk**	2 (7.7)	13 (50.0)	11 (42.3)	0.554	0.152
**Risk**	2 (8.0)	16 (64.0)	7 (28.0)		
	**UBS**				
**No risk**	0 (0)	15 (57.7)	11 (42.3)	0.475	0.171
**Risk**	1 (4.0)	16 (64.0)	8 (32.0)		
	**PC**				
**No risk**	0 (0)	15 (57.7)	11 (42.3)	0.293	0.219
**Risk**	2 (8.0)	15 (60.0)	8 (32.0)		
	**BES**				
**DOS**	**Low**	**Average**	**High**	***p*-value**	**V Cramer**
	**PA**				
**No risk**	3 (10.0)	20 (66.7)	7 (23.3)	0.307	0.217
**Risk**	1 (5.9)	7 (41.2)	9 (52.9)		
**Presence**	0 (0)	2 (50.0)	2 (50.0)		
	**UBS**				
**No risk**	0 (0)	21 (70.0)	9 (30.0)	0.398	0.199
**Risk**	1 (5.9)	8 (47.1)	8 (47.1)		
**Presence**	0 (0)	2 (50.0)	2 (50.0)		
	**PC**				
**No risk**	1 (3.3)	20 (66.7)	9 (30.0)	0.708	0.145
**Risk**	1 (5.9)	8 (47.1)	8 (47.1)		
**Presence**	0 (0)	2 (50.0)	2 (50.0)		

BES: Body Esteem Scale; DOS: Düsseldorf Orthorexia Scale; PA: Physical Attractiveness; UBS: Upper Body Strength; PC: Physical Condition

## Discussion

EDs among elite soccer players are an issue, conditioned by the high-pressure competitive sports environment and the emphasis on physical fitness. Factors contributing to the emergence of EDs among this group include aesthetic expectations in sports, cultural emphasis on thinness as an attribute of fitness, and mental stress of participating in elite competitions.

The consequences of EDs can be severe and multifaceted, encompassing both the mental well-being and physical performance of athletes. Physiological effects of inadequate nutrition include muscle weakness, fatigue, injury, and a decline in overall fitness. Psychologically, an obsessive focus on diet, weight, and the body shape can lead to anxiety, depression, and burnout, negatively affecting the longevity of a sports career.

This study was conducted to assess the risk of EDs and analyze self-perceptions regarding body composition among elite soccer players. The analysis aimed to provide valuable insight into the challenges elite soccer players face in maintaining a healthy relationship with food and body image. By identifying the magnitude of the problem and factors contributing to the risk of developing EDs, there is an opportunity to target interventions and support systems tailored to the unique needs of elite athletes.

The results indicate the prevalence of ED risk among elite soccer players, with more than 51% of respondents exhibiting characteristics indicative of this risk. In addition, a relationship was observed between elements of body composition and the EAT-26 test score; players with greater body fat content were characterized by a higher risk of EDs. A study by Godoy-Izquierdo et al. (2019) analyzed the prevalence of EDs among athletes including soccer players using the Cuestionario de Hábitos Alimentarios del Deportista (CHAD). That study found that 11.4% of soccer players were at risk of developing or having EDs and it highlighted the higher risk of the disorder in this group, reaching more than 50%. The above differences may be due to a different survey instrument used in the study or cultural differences, as the participants were of the Spanish nationality (Godoy-Izquierdo et al., 2019). The study by Abbott et al. (2021) used the EAT-26 questionnaire, and the study participants consisted of elite male soccer players (n = 137), female soccer players (n = 70), and non-athletes (179). Soccer players had a higher EAT-26 score than control subjects (10.4 ± 9.9 vs. 6.8 ± 6.7; *p* = 0.001). There were no differences in the incidence of risk between the groups; the risk of developing EDs affected about 15% of the subjects (Abbott et al., 2021). As in the previous case, our results showed a greater magnitude of the problem, what is interesting considering that the same questionnaire was used. Baldó Vela et al. (2021) determined the prevalence of EDs in adult male team sports players (n = 124), using the EAT-40 questionnaire. Their results indicated that 18.5% of the subjects had a risk of developing EDs (Baldó Vela et al., 2021). Gouttebarge et al. (2017) studied the prevalence and the incidence of symptoms of common mental disorders including EDs among professional soccer referees (n = 391) using self-report questionnaires. They showed that EDs affected up to 29% of the subjects (Gouttebarge et al., 2017). A study by Staśkiewicz-Bartecka and Kardas (2024) on amateur and professional soccer players, using the EAT-26 questionnaire, found that the risk of EDs was about 17% in both groups of subjects. The use of social media was linked to the incidence of EDs, which is a significant problem today (Staśkiewicz-Bartecka and Kardas, 2024). McDonald et al. (2020) conducted a study to examine gender differences in EAT-26 scale scores among athletes participating in National Collegiate Athletic Association (NCAA) “slimming” and “non-slimming” sports (n = 121). Their results showed a significant impact of the type of the practiced sport on attitudes and behaviors related to nutrition. Male athletes in “non-slimming” sports scored higher in the attitudes section, while male athletes in “slimming” sports scored higher in the eating behavior section. In addition, female athletes, regardless of the type of the sport they practiced, achieved similar scores in both sections of the EAT-26. That study indicates that athletes, regardless of gender or the type of sport, may exhibit symptoms of EDs, and gender differences may be smaller in the athletes’ population. The above results indicate the prevalence of the ED problem among athletes including soccer players and people associated with the sport, however, the scale of the problem shown in our study is much higher when compared to the results of other studies (McDonald et al., 2020).

The risk of ON in our study was found in about 1/3 of athletes. About 8% of soccer players, according to the interpretation of the DOS, were found to have ON. In the available literature, there are few studies evaluating the risk of ON in soccer players. A study by Yıldız et al. (2020) assessed the risk of ON in a group of sedentary individuals, youth soccer players, and female soccer players (n = 101). The ORTO-15 questionnaire was used in their study, and the results showed that the mean score of the ORTO-15 scale was higher in the women's group. Moreover, the mean number of points scored by soccer players was higher than by individuals with a sedentary lifestyle (Yildiz et al., 2020). Surała et al. (2020) evaluated the prevalence of ON traits and their relationship to body composition among athletes of various sports (n = 273) using the ORTO-15 questionnaire. Depending on the cutoff values applied, the ON tendency was manifested by 41.3% to 88.3% of the subjects. That study did not show differences between genders or the activity type, although the ORTO-15 score was associated with body weight, the BMI, and visceral fat in the male group (Surała et al., 2020). A study by Seguara-Garcia et al. (2012) also used the ORTO-15 and EAT-26 questionnaires and their study group consisted of Italian athletes and a control group. It was found that about 28% of athletes had a positive ORTO-15 score and 14% had a risk of EDs according to the EAT-26 results’ interpretation (Segura-García et al., 2012). Those authors indicated an association between the ON risk and EDs, which was not supported by our study results. The present study also found no correlation between ON trends and the BMI in soccer players. The effect of the BMI on ON risk has been extensively studied in other populations, yet the results are inconsistent. Several studies have shown that ON was associated with the BMI (Bundros et al., 2016; Oberle and Lipschuetz, 2018). In most studies, the authors observed no correlation between ON and BMI classification (Agopyan et al., 2019; Melin et al., 2019). The relationship of body weight and body composition with ON risk was analyzed by Agopyan et al. (2019) in a study using the ORTO-11 and EAT-40 questionnaires. Their study group consisted of female dietetics students (n = 136). It was found that 70.6% of female students were at risk for ON, although no correlation was identified between the ORTO-11 and the EAT-40 or body composition (Agopyan et al., 2019).

Soccer players rated their body image moderately or highly in each of the three sub-scales analyzed. A correlation was found between the body fat percentage and upper body strength ratings; players with lower body fat rated their bodies higher in this category. A study by Staśkiewicz-Bartecka and Kardas (2024) assessed the body self-assessment of amateur and professional athletes using the BES. Differences were found between the athletic level and body evaluation of soccer players, with professional players rating their bodies higher than amateurs. Amateurs had moderate to low body ratings in all three subscales, while professional players had moderate to high body ratings. The present results confirm the findings of that study (Staśkiewicz-Bartecka and Kardas, 2024). Kong and Harris (2015) conducted an analysis comparing body dissatisfaction and ED symptoms among 320 athletes, both at the elite and amateur levels. The study group consisted of subjects practicing sports that either required maintaining a slim physique or did not place as much emphasis on it. It was found that regardless of whether they were high-level or recreational athletes, those who practiced sports that emphasized a slim physique were more likely to express dissatisfaction with their bodies and more likely to experience symptoms of EDs. Athletes in the upper leagues reported greater body dissatisfaction and greater ED symptomatology regardless of the type of the practiced sport, while no such differences were observed among amateur and non-athletes. In addition, it was reported that more than 60% of elite athletes from both types of sports experienced pressure from coaches regarding their body image (Kong and Harris, 2015).

Our study conducted on elite soccer players has several strengths. We used a broad set of research tools including EAT-26, DOS, and BES standardized questionnaires along with DSM-BIA body composition analysis providing a multi-faceted assessment of the subjects under study. In addition, the survey was conducted during the same period of the soccer season, minimizing potential variability due to seasonal training differences. With a response rate of 94.4%, the survey ensures a high level of participants’ involvement and data representativeness. The inclusion of players of different nationalities and leagues contributes to the diversity of the sample, which may increase the generalizability of the results in the context of professional soccer. In addition, the survey defined specific criteria for selecting participants, which clarifies the target population and increases the replicability of the study.

However, the present study also has some limitations. With only 51 participants, the results may not be generalizable to all soccer players or athletes from other sports and levels. Moreover, the dedicated sampling used, while purposeful, may not reflect the broader population of soccer players or the impact of unmeasured confounding variables. This study was conducted only among men, which does not allow for an examination of possible gender differences. The self-assessment methods used may not be fully objective, as they are based on the subjective evaluation of participants. However, during the study, appropriate procedures were implemented to ensure the anonymity of participants to reduce the pressure of public perception, the objectives of the study were clearly stated, and honest and open answers were encouraged.

## Conclusions

The results of our study on ED risk among elite soccer players showed that more than half of the players were at risk for developing the disorder, highlighting a concerning prevalence within this population. Players with higher muscle mass content presented a greater risk in relation to attitudes toward eating. In contrast, greater fat mass content was associated with a higher risk considering behavioral questions. Higher body fat content was found to be associated with a higher risk of developing EDs. The analysis of the DOS questionnaire indicated that approximately one-third of the athletes were at risk of developing ON, with nearly 10% currently experiencing ON. However, no significant relationship was found between body composition and the risk of ON among elite soccer players. Regarding body perception, elite soccer players generally displayed a moderate assessment of their bodies. They rated physical fitness, sex drive, health, and sexual activity issues highly, while feet and body hair were rated lowest. Notably, higher body fat content was linked to lower upper body strength scores, indicating a potential impact on athletes' perceptions of their physical capabilities.

Importantly, no correlation was identified between the risk of EDs and ON and body esteem, suggesting that self-perception concerning body image may not directly influence the likelihood of developing these disorders among elite soccer players. Overall, these findings underscore the need for targeted interventions and support systems to address the high prevalence of eating disorders among elite soccer players and promote positive body image and overall well-being within this population.
